# Clinical significance of increased arterial stiffness associated with atrial fibrillation, according to Framingham risk score

**DOI:** 10.1038/s41598-021-84311-9

**Published:** 2021-03-02

**Authors:** Goh Eun Chung, Hyo Eun Park, Heesun Lee, Su-Yeon Choi

**Affiliations:** 1 Division of Gastroenterology, Department of Internal Medicine, Healthcare System Gangnam Center, Seoul National University Hospital, Seoul, Korea; 2 Division of Cardiology, Department of Internal Medicine, Healthcare System Gangnam Center, Seoul National University Hospital, 39FL. Gangnam Finance Center 737, Yeoksam-Dong, Gangnam-Gu, Seoul, 135-984 Korea

**Keywords:** Cardiology, Risk factors

## Abstract

Atrial fibrillation (AF) is the most common arrhythmia in the elderly. Arterial stiffness may predict the risk of AF, but this relationship has not been fully evaluated. We assessed the association between arterial stiffness and prevalent AF. All subjects who had electrocardiography performed and a cardio-ankle vascular index (CAVI) calculated during a screening examination between 2010 and 2019 were enrolled. To evaluate the association between increased arterial stiffness and AF, we divided the population according to their Framingham risk score (FRS) into low-, intermediate-, and high-risk groups. A total of 8048 subjects were evaluated. The multivariate analysis revealed that increased arterial stiffness was significantly associated with AF prevalence, even after adjusting cardiovascular risk factors [odds ratio (OR) 1.685, 95% confidence interval (CI) 1.908–2.588, *p* = 0.017]. When we subcategorized the subjects according to their FRS, increased arterial stiffness was significantly associated with AF in the intermediate- and high-risk groups (OR 3.062, 95% CI 1.39-6.740 and OR3.877, 95% CI 1.142-13.167, respectively, BMI adjusted. High arterial stiffness shows a significant association with AF in those with intermediate or high cardiovascular risk, and can be used for further risk stratification of patients.

## Introduction

Arterial stiffness is a parameter that reflects the progression of atherosclerosis^1–3^. It increases with age and predicts coronary heart disease, stroke, and mortality^4^. Traditional cardiovascular risk factors (age, gender, smoking, obesity, hypertension, diabetes mellitus, and dyslipidemia) are also known to be risk factors for increased arterial stiffness and have shown an association with atrial fibrillation (AF)^5–7^. Both the incidence and prevalence of AF have gradually increased in Korea^8^, due primarily to lifestyle westernization and associated comorbidities, as well as to an aging population. With the increasing incidence of AF, related adverse outcomes are becoming an important concern in Korean society, leading to significant medical costs and a poor quality of life. As AF increases the risk of adverse cardiovascular and cerebrovascular events^9–11^, much effort has been focused on stroke prevention and modification of risk factors^12,13^.

The Framingham risk score (FRS) has been used to predict a patient’s relative risk for cardiovascular events^14–16^. The FRS is derived from a mathematical algorithm using traditional risk factors as weighted variables and has been used as the foundation of risk stratification for the prediction of cardiovascular events. The Framingham Heart Study and Multi-Ethnic Study of Atherosclerosis suggested peripheral pulse pressure as a risk factor for incident AF^3,17,18^, whereas another study showed that impaired vascular function in AF was mediated by age and classic risk factors of atherosclerosis, suggesting that noninvasive vascular function measures do not improve the discriminatory ability of AF^6^. Arterial stiffness and vascular function can be assessed by multiple measures, classically flow mediated dilation, augmentation index, and pulse wave velocity. In our study we have used cardio-ankle vascular index (CAVI), which has an important advantage over other parameters, as CAVI is independent from blood pressure^19,20^. Recently a causal link was suggested between PWV and AF, Currently, the association between arterial stiffness and AF is inconclusive^6,21^, and it is unknown whether the significance of arterial stiffness differs according to relative cardiovascular risk in association with AF.

In this study, we evaluated the association between arterial stiffness and AF in the general population without known cardiovascular disease, according to the FRS.

## Methods

### Study population

**Figure 1:**
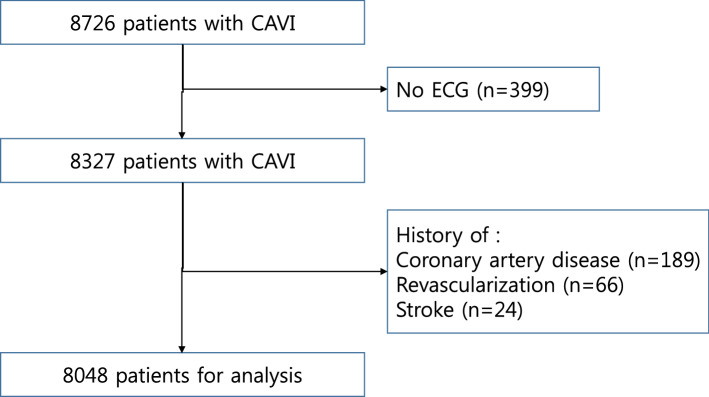
Inclusion and exclusion of the study population.

The current study was performed as a retrospective cross-sectional study. All apparently healthy subjects with a 12-lead electrocardiogram (ECG) and a cardio-ankle vascular index (CAVI) on the same day during a health check-up examination at the Healthcare System Gangnam Center, Seoul National University Hospital, between January 2010 and December 2019, were screened for enrollment in this study. Health examination has become popular in Korea because a thorough medical checkup can be performed in a few hours and the majority of referred hospitals in Korea is now equipped with a healthcare center to provide check-ups for screening purpose. The subjects voluntarily attended for a general health check-up, while others were supported by their employer. Among 8726 patients with a CAVI, those without an ECG (n = 399), with a history of angina (n = 189), or with a previous percutaneous coronary intervention (n = 66) or stroke (n = 24) were excluded, leaving 8048 subjects to be included in the analysis (Fig. 1).

Information including past medical history, comorbidities, and medications were obtained using subject-recorded questionnaires and confirmed during the CAVI measurement. Patients were asked about their past history of cardiovascular disease, cerebrovascular disease, peripheral arterial disease, hypertension, diabetes mellitus, dyslipidemia, gout, and chronic kidney disease. Additionally, patients were asked about their smoking and alcohol use. The risk score for cardiovascular disease was determined using the FRS^22^.

The study protocol followed the Declaration of Helsinki of 1975, as revised in 1983. This study was approved by the Institutional Review Board of Seoul National University Hospital (H-1912-114-1090). Because the current study was performed using a retrospective design (use of a database and medical records), informed consent was waived by the board.

### Anthropometric and laboratory parameters

The measurements of anthropometric and laboratory parameters were performed on the day of the health check-up examination. Blood pressure, body weight, and height were measured. The body mass index (BMI) was calculated using height and body weight measured using a digital scale, according to the formula: BMI = weight (kg)/height (m^2^).

Blood tests were performed after at least 12 h of fasting, which included levels of total cholesterol, triglyceride (TG), high-density lipoprotein (HDL)-cholesterol, low-density lipoprotein (LDL)-cholesterol, fasting blood sugar (FBS), glycosylated hemoglobin (HbA1c), hemoglobin, uric acid, blood urea nitrogen, and serum creatinine. Measurements of LDL-cholesterol were used for analyses. An ECG was also obtained on the same day as the health check-up with the patient in the supine position, using 10 electrodes placed on the four limbs and chest surface, over a duration of 10 s and read by three different cardiologists.

### Assessment of arterial stiffness using the CAVI

The CAVI was measured using the VaSera VS-1000 (Fukuda Denshi Co. Ltd, Tokyo, Japan), as described previously^19,23–25^. Each patient was placed in a sitting position for at least 5 min, and the brachial pulse pressure was measured using an automatic cuff oscillometric device. Cuffs were applied to the four extremities (upper arms and ankles), with patients in the supine position. After resting for 10 min, the measurement was performed. The systolic, diastolic, and pulse pressures were measured twice with the patients in the supine position, and an average value was taken. A phonocardiography sensor was placed at the right sternal border in the second intercostal space, and ECG leads were attached to both wrists. The CAVI was determined using the following equation:

$${\text{CAVI }} = {\text{ a}}[\left( {{2}\rho / \, \Delta {\text{P}}} \right) \times {\text{ln}}\left( {{\text{Ps }}/{\text{ Pd}}} \right) \times {\text{PWV}}^{{2}} ] \, + {\text{ b}}$$

with Ps and Pd as the systolic and diastolic blood pressures, respectively, PWV as the pulse-wave velocity, ΔP as Ps – Pd, *ρ* as the blood density, and a and b as constants. For analysis, the mean value of the left and right indices was calculated, and the patients were grouped into a CAVI greater than or equal to 8 or a CAVI less than 8. A CAVI greater than or equal to 8 was defined as increased arterial stiffness, which has been shown to be associated with significant coronary artery stenosis, calcifications, and adverse cardiovascular events in previous studies^24–26^.

### Statistical analysis

Data are expressed as the mean ± standard deviation for continuous variables and as frequencies for categorical variables. Chi-square tests and Student’s *t* tests were used for categorical and continuous variables, respectively, to compare the differences between groups. The univariate and multivariate logistic regression analyses were performed to assess the association between increased arterial stiffness and AF, and multiple models were evaluated to adjust for possible confounders. Among variables with a *p* value < 0.05 in univariate analyses, those with clinical importance were subjected to multivariate analyses. Confounding variables with potentially multiple collinearity problems were excluded. To evaluate the association between increased arterial stiffness and AF, according to the FRS, patients were categorized into three groups: low-, intermediate-, and high-risk, and the significance of high arterial stiffness was determined for each group. All statistical analyses were performed using the Statistical Package for Social Science software (IBM SPSS Statistics, version 23), and two-tailed *p* values less than 0.05 were considered to be statistically significant.

## Results

### Baseline characteristics

As shown in Table 1, patients with high arterial stiffness were older (62 ± 9 years vs. 53 ± 8 years, *p* < 0.001, CAVI ≥ 8 vs. CAVI < 8, respectively). In patients with a CAVI greater than or equal to 8, hypertension (*p* = 0.000), diabetes mellitus (*p* < 0.001), and dyslipidemia (*p* < 0.001) were more common than in patients with a CAVI less than 8. Systolic and diastolic blood pressures (both *p* < 0.001) and levels of FBS and HbA1C (*p* < 0.001 for both) were all higher in patients with a CAVI ≥ 8 than in patients with a CAVI < 8. AF was more frequently found in patients with a CAVI ≥ 8 than in those with a CAVI < 8 (2.2% vs. 1.0%, respectively, *p* < 0.001).

**Table 1:** Baseline characteristics according to arterial stiffness.

Parameters	CAVI ≥ 8 (n = 3344)	CAVI < 8 (n = 4704)	p-value
Age, years	62 ± 9	53 ± 8	< 0.001
Male gender, n (%)	2393 (71.6%)	3122 (66.4%)	< 0.001
Body mass index, kg/m^2^	24 ± 3	24 ± 4	< 0.001
Waist circumference, cm	87 ± 8	86 ± 9	< 0.001
**Comorbidity, n (%)**			
Hypertension	1276 (38.2%)	998 (21.2%)	< 0.001
Diabetes mellitus	548 (16.4%)	275 (5.8%)	< 0.001
Dyslipidemia	947 (28.3%)	798 (17.0%)	< 0.001
Current smoking	572 (17.5%)	873 (19.0%)	0.081
Framingham risk score			< 0.001
Low (< 10%)	1549 (46.5%)	3494 (74.4%)	
Intermediate (10–20%)	998 (29.9%)	779 (16.6%)	
High (> 20%)	786 (23.6%)	425 (9.0%)	
Systolic blood pressure, mmHg	132 ± 25	125 ± 21	< 0.001
Diastolic blood pressure, mmHg	85 ± 10	82 ± 9	< 0.001
Hemoglobin, g/dL	14.7 ± 1.3	14.6 ± 1.4	0.001
Blood urea nitrogen, mg/dL	15.2 ± 4.1	14.2 ± 3.6	< 0.001
Creatinine, mg/dL	0.87 ± 0.20	0.86 ± 0.28	0.001
Fasting blood sugar, mg/dL	110 ± 27	101 ± 18	< 0.001
HbA1C, %	6.0 ± 0.9	5.7 ± 0.6	< 0.001
Total cholesterol, mg/dL	192 ± 37	197 ± 35	< 0.001
Triglyceride, mg/dL	126 ± 78	121 ± 79	0.002
HDL cholesterol, mg/dL	55 ± 16	56 ± 15	0.667
LDL cholesterol, mg/dL	120 ± 32	125 ± 31	< 0.001
Uric acid, mg/dL	5.7 ± 1.4	5.7 ± 1.4	0.711
hsCRP, mg/dL	0.54 ± 1.62	0.51 ± 1.63	0.510
AF on electrocardiogram	73 (2.2%)	45 (1.0%)	< 0.001

*AF* atrial fibrillation, *CAVI* cardio-ankle vascular index, *HDL* high-density lipoprotein, *hsCRP* high-sensitivity C-reactive protein, *HbA1C* glycated hemoglobin, *LDL* low-density lipoprotein.

### Associations between different parameters and the prevalent AF

To evaluate the association between each parameter and the prevalent AF, univariate analysis was performed. An age greater than or equal to 65 years (odds ratio (OR) 2.606, 95% confidence interval (CI) 1.787–3.801, *p* = 0.000), male gender (OR 3.458, 95% CI 1.975–6.054, *p* < 0.001), BMI greater than or equal to 25 kg/m^2^ (OR 1.529, 95% CI 1.060–2.205, *p* = 0.023), and FBS level (OR 1.010, 95% CI 1.005–1.015, *p* = 0.000) were significantly associated with the prevalent AF. Comorbidities, including hypertension (*p* = 0.479), diabetes mellitus (*p* = 0.397), dyslipidemia (*p* = 0.210), and smoking (*p* = 0.245), were not associated with the prevalent AF. A CAVI greater than or equal to 8 (OR 2.311, 95% CI 1.589–3.359, *p* < 0.001) was associated with the prevalent AF (Table 2).

**Table 2:** Univariate analysis for association with the prevalent AF.

	OR	95% CI	p-value
Age ≥ 65	2.606	1.787–3.801	< 0.001
Male gender	3.458	1.975–6.054	< 0.001
Body mass index ≥ 25	1.529	1.060–2.205	0.023
Waist circumference, cm	1.040	1.018–1.062	0.000
Hypertension	2.042	0.283–14.724	0.479
Diabetes mellitus	2.348	0.326–16.917	0.397
Dyslipidemia	0.734	0.452–1.191	0.210
Smoking	0.729	0.428–1.241	0.245
Systolic blood pressure, mmHg	1.002	0.998–1.006	0.405
Diastolic blood pressure, mmHg	1.025	1.007–1.044	0.006
Hemoglobin, g/dL	1.420	1.234–1.634	< 0.001
Blood urea nitrogen, mg/dL	1.069	1.035–1.104	< 0.001
Creatinine, mg/dL	1.409	1.070–1.855	0.014
Fasting blood sugar, mg/dL	1.010	1.005–1.015	< 0.001
HbA1C, %	1.093	0.878–1.360	0.425
Total cholesterol, mg/dL	0.993	0.988–0.998	0.007
Triglyceride, mg/dL	1.000	0.997–1.002	0.710
HDL cholesterol, mg/dL	0.996	0.983–1.008	0.478
LDL cholesterol, mg/dL	0.993	0.987–0.999	0.022
Uric acid, mg/dL	1.263	1.116–1.430	< 0.001
hsCRP, mg/dL	1.045	0.935–1.169	0.438
CAVI ≥ 8	2.311	1.589–3.359	< 0.001

*AF* atrial fibrillation, *CAVI* cardio-ankle vascular index, *HDL* high-density lipoprotein, *hsCRP* high-sensitivity C-reactive protein, *HbA1C* glycated hemoglobin, *LDL* low-density lipoprotein.

Multivariate models were evaluated for the adjusted risk of increased arterial stiffness in relation to the prevalent AF (Table 3). Model I included age greater than or equal to 65 years, gender, presence of hypertension and diabetes mellitus, and LDL-cholesterol level greater than or equal to 130 mg/dL. Model II included age greater than or equal to 65 years, gender, BMI greater than or equal to 25 kg/m^2^, smoking, presence of hypertension and diabetes mellitus, and LDL-cholesterol level greater than or equal to 130 mg/dL. Model III included the FRS for adjustment. A CAVI greater than or equal to 8 was the most significant parameter associated with the prevalent AF in all models (adjusted OR 1.642, 95% CI 1.082–2.492, *p* = 0.020 for Model I; adjusted OR 1.685, 95% CI 1.908–2.588, *p* = 0.017 for Model II; and adjusted OR 2.064, 95% CI 1.397–3.050, *p* < 0.001 for Model III).

**Table 3:** Multivariate models for association between arterial stiffness and the prevalent AF.

Parameters	Odds ratio	95% Confidence interval	p-value
**Model I**			
Age ≥ 65	2.049	1.334–3.145	0.001
Male gender	3.455	1.966–6.071	0.000
Hypertension	1.428	0.967–2.110	0.073
Diabetes mellitus	0.780	0.442–1.376	0.391
LDL cholesterol ≥ 130 mg/dL	0.870	0.584–1.296	0.493
CAVI ≥ 8	1.642	1.082–2.492	0.020
**Model II**			
Age ≥ 65	1.941	1.245–3.028	0.003
Male gender	3.585	1.99–6.497	< 0.001
BMI ≥ 25	1.302	0.883–1.919	0.183
Smoking	0.634	0.368–1.093	0.101
Hypertension	1.282	0.852–1.930	0.234
Diabetes mellitus	0.775	0.431–1.394	0.394
LDL cholesterol ≥ 130 mg/dL	0.855	0.568–1.287	0.452
CAVI ≥ 8	1.685	1.098–2.588	0.017
**Model III**			
FRS Intermediate risk	1.661	1.087–2.538	0.019
FRS high risk	1.353	0.817–2.243	0.240
CAVI ≥ 8	2.064	1.397–3.050	< 0.001

*BMI* body mass index, *CAVI* cardio-ankle vascular index, *FRS* Framingham risk score, *LDL* low density lipoprotein.

### Differences in significance of increased arterial stiffness according to the FRS

Model III revealed that even after adjusting for cardiovascular risk using the FRS, a CAVI greater than or equal to 8 was still a significant parameter in the prevalent AF. The study subjects were categorized into three groups-low, intermediate, and high risk of cardiovascular events-according to the FRS, and each group was evaluated separately. High arterial stiffness was not an equally significant parameter related to the prevalent AF in the three groups. A CAVI greater than or equal to 8 was a significant parameter in subjects with an intermediate or high risk of cardiovascular events, according to the FRS (OR 3.094, 95% CI 1.414–6.769, *p* = 0.005 for the intermediate-risk group; OR 3.690, 95% CI 1.090–12.490, *p* = 0.036 for the high-risk group).

Since the FRS is derived from an algorithm that includes traditional cardiovascular risk factors (age, gender, blood pressure, cholesterol levels, smoking behavior, and diabetes status), we performed additional adjustments for a BMI greater than or equal to 25 kg/m^2^ to evaluate the significance of a CAVI greater than or equal to 8 in the different risk groups (Table 4). A CAVI greater than or equal to 8 still showed a significant association with the prevalent AF in both the intermediate- and high-risk groups (adjusted OR 3.062, 95% CI 1.391–6.740, *p* = 0.005 for the intermediate-risk group; adjusted OR 3.877, 95% CI 1.142–13.167, *p* = 0.030 for the high-risk group). In the low-risk group, a CAVI greater than or equal to 8 was not a significant parameter in either the unadjusted or adjusted model (OR 1.465, 95% CI 0.854–2.513, *p* = 0.166 for the unadjusted model; OR 1.535, 95% CI 0.892–2.640, *p* = 0.122 for the adjusted model).

**Table 4:** Association between CAVI ≥ 8 and the prevalent AF according to FRS.

Parameters	Odds ratio	95% Confidence interval	p-value
**Unadjusted**			
Low risk group			
CAVI ≥ 8	1.465	0.854–2.513	0.166
Intermediate risk group			
CAVI ≥ 8	3.094	1.414–6.769	0.005
High risk group			
CAVI ≥ 8	3.690	1.090–12.490	0.036
**Adjusted with BMI ≥ 25**			
Low risk group			
CAVI ≥ 8	1.535	0.892–2.640	0.122
Intermediate risk group			
CAVI ≥ 8	3.062	1.391–6.740	0.005
High risk group			
CAVI ≥ 8	3.877	1.142–13.167	0.030

*BMI* body mass index, *CAVI* cardio-ankle vascular index.

## Discussion

In this study, the prevalence of AF was higher in patients with high arterial stiffness, as determined by the CAVI. The significance of a CAVI greater than or equal to 8 differed according to the relative risk of cardiovascular events, as measured by the FRS. High arterial stiffness showed a significant association with the prevalent AF in those with an intermediate or high cardiovascular risk, whereas it was not a significant parameter in the low-risk group.

One of the strengths of our study is that our patient population represents an apparently healthy general adult population of a homogeneous ethnicity. All measured parameters were used for screening purposes in healthy subjects without known cardiovascular disease; thus, our data can be used for primary prevention in the general population. An interpretation of high arterial stiffness without known cardiovascular risk factors or any cardiac symptoms can be a challenge for physicians during medical screening, especially in those with a low or intermediate FRS. Our previous studies have tried to identify those individuals at relatively high risk for coronary atherosclerosis using the CAVI^24,25,27^, and we have tried to develop a more tailored and comprehensive approach for managing each person’s cardiovascular risk. In this study, we evaluated the usefulness and significance of a CAVI greater than or equal to 8, according to the FRS, in relation to the prevalent AF.

There is a need for an accurate and reliable estimate of absolute cardiovascular disease risk in order to identify those in need of aggressive medical management. Using multiple variables, the FRS plays an important role in the identification of at-risk patients and can be used as the foundation for modifying risk score models. Although widely used, the FRS has well-known limitations: Shaw et al. showed that 45% of a group at intermediate risk for cardiovascular events were shifted to the high-risk group when the FRS was re-calculated using coronary artery calcium-adjusted age^28^. Thus, cardiovascular risk cannot be solely evaluated using the FRS, especially in those at low or intermediate risk for cardiovascular events, and subjects with a relatively high risk need to be re-stratified for follow-up or appropriate medical service. As shown in our study, subjects with an intermediate or high FRS must be monitored and screened for not only coronary artery disease but also for the prevalent AF because if underdiagnosed, AF may present as cerebrovascular disease and cause significant morbidity, mortality, and an extremely poor quality of life.

Current evidence suggests that arterial stiffness reflects the integrated effects of traditional cardiovascular risk factors on vessels, as endothelial dysfunction is an early event in the development and progression of atherosclerotic disease. Vascular stiffening is also associated with risk factors of AF, and high pulse pressure is known to be a risk factor for incident AF^3,29^. Moreover, arterial stiffness is known to affect recurrence rates of AF after elective cardioversion^30^. Impaired vascular function has shown improvement after restoration of sinus rhythm in patients with AF^31^. Among various parameters used to measure arterial stiffness, the CAVI is a simple, effective, and reliable parameter used for screening arterial stiffness at many medical screening centers in Korea: It reflects the stiffness of whole arterial segments from the aorta to the tibial artery and is known to be a superior index of arterial stiffness compared to traditionally established parameters, such as brachial ankle pulse-wave velocity^32^. The CAVI is also independent of blood pressure, unlike other parameters^33^. The reliable measurement of arterial stiffness in AF has been evaluated in another study by Caluwe et al., and although that study did not use the CAVI, its results showed similar to our study^34^.

Although our study does not focus on anticoagulation therapy in patients diagnosed with AF, the clinical significance of high arterial stiffness in association with the prevalent AF provides an additional method for further risk stratification among those who may have been categorized into a low-risk group according to the CHA_2_DS_2_-VASc score. Anticoagulation therapy for stroke prevention in patients with AF is based on risk stratification using the CHA_2_DS_2_-VASc score, but the indications for anticoagulation therapy in patients with low scores are still vague. The 2016 guidelines from the European Society of Cardiology recommend an individualized approach for the weighting of risk factors^35^. The guidelines also suggest that biomarker-based risk scores may help better stratify patients, and, as shown in our study, arterial stiffness measured by the CAVI may serve as a marker to identify those who need early anticoagulation treatment, when more prognostic data is available.

### Limitations

Our study has some limitations. First, as the study was designed as a retrospective review of medical records, the causal relationship between increased arterial stiffness and incident AF cannot be evaluated. Second, we also do not have data regarding future cardiovascular events in study subjects, so the prognostic value of our findings can only be elucidated in future studies. From a previous study, high pulse-wave velocity was associated with increased cardiovascular events and improved the prediction of adverse cardiovascular events in patients with AF^36^. As we have suggested above, we have found that the association between AF and high arterial stiffness differs according to the relative cardiovascular risk of each patient; thus, arterial stiffness can help improve risk stratification. Although we cannot provide guidelines for treatment measures to prevent cardiovascular events or incident AF, we provide a tool to define those who should be monitored and managed for cardiovascular risk factors more aggressively, even within the same FRS group. Future studies with serial CAVI and ECG data may help identify the incidence and associated parameters of AF. Third, it is possible that there may be a selection bias that the majority of participants of the study population interested in health care were included in the study. Lastly, there are potential missed AF patients for whom no AF episodes were recorded during the ECG evaluation, such as paroxysmal AF or those with sinus conversion after treatment.

## Conclusion

The clinical significance of a high CAVI in relation to AF differs according to the FRS. These findings imply that arterial stiffness can be used for risk stratification of patients with AF, especially those with an intermediate or high risk for cardiovascular events.
